# Neurosymptomatic carvenous sinus meningioma: a 15-years experience with fractionated stereotactic radiotherapy and radiosurgery

**DOI:** 10.1186/1748-717X-9-27

**Published:** 2014-01-17

**Authors:** Sebastião Francisco Miranda Correa, Gustavo Nader Marta, Manoel Jacobsen Teixeira

**Affiliations:** 1 Radiation Oncology Department - Hospital Sírio-Libanês, Rua Dona Adma Jafet, 91 - Bela Vista, CEP 01308-050 Sao Paulo, SP, Brazil; 2 Radiation Oncology Department Instituto do Câncer do Estado de São Paulo (ICESP), Sao Paulo, Brazil; 3 Department of Neurology at the Medical School, University of São Paulo, São Paulo, SP, Brazil

**Keywords:** Meningioma, Radiotherapy, Radiosurgery, Fractionated stereotactic, Radiotherapy

## Abstract

**Background:**

The tumor removal of Cavernous Sinus Meningiomas usually results in severe neurological deficits. Stereotactic radiosurgery (SRS) and fractionated Stereotactic radiotherapy (SRT) are advanced modalities of radiotherapy for treatment of patients with inoperable and symptomatic CSMs. The authors evaluated the long term symptomatology, the image findings, and the toxicity of patients with CSMs treated with SRS or SRT.

**Patients and methods:**

From 1994 to 2009, 89 patients with symptomatic CSMs were treated with SRS or SRT. The indication was based on tumour volume and or proximity to the optic chiasm. The median single dose of SRS was 14 Gy, while the SRT total dose, ranged from 50.4 to 54 Gy fractionated in 1.8-2 Gy/dose. The median follow-up period lasted 73 months.

**Results:**

The clinical and radiological improvement was the same despite the method of radiotherapy; 41.6% (SRS) and 48.3% (SRT) of patients treated. The disease-free survivals were 98.8%, 92.3% and 92.3%, in 5, 10, and 15 years, respectively. There was no statistical difference in relation to the symptoms and image findings between both methods. According to the Common Toxicity Criteria, 7% of the patients presented transient optic neuropathy during 3 months (grade 2) and recovered with dexamethasone, 2 patients had trigeminal neuropathy (grade 2) and improved rapidly, and one patient presented total occlusion of the internal carotid artery without neurological deficit (grade 2). Temporary lethargy and headache (grade 1) were the most frequent immediate complications. No severe complications occurred.

**Conclusions:**

Stereotactic Radiosurgery and fractionated Stereotactic Radiotherapy were equally safe and effective in the management of symptomatic CSMs.

## Background

Meningiomas account for 13% to 26% of all intracranial neoplasms. Cavernous sinus meningiomas (CSMs) occur in 0.5 per 100,000 persons in the general population. The vast majority of meningiomas are benign, well differentiated, and with low proliferative potential. The most common clinical features of meningiomas are neurological deficits (e.g. amblyopia), epilepsy, and headache. However, there are an increasing number of asymptomatic patients with CSMs because CT scans or MR is commonly used for evaluation of other medical conditions, as cranial trauma and allows the diagnosis in the preclinical phase. Histological type is the major predictor of meningioma behavior [1].

Despite technical advances regarding microsurgical resections of cavernous sinus meningiomas, they are rarely completely resected and are often accompanied by a high rate of neurological disturbances. After partial or subtotal tumour removal, the probability of recurrence remains significant (13% at 3 years; 38% at 5 years) [2].

The treatment of CSMs aims the best survival and local control coupled with the least possible morbidity. It includes close observation, surgical resection, radiotherapy, systemic therapy or a combination of these approaches. Management decisions obviously should have to take into account the patient-related factors (age, performance status, co-morbidities, and symptoms), and tumor features (size, localization, and histological grade) [1,3]. Given the high incidence of local recurrence, radiotherapy usually is indicated when surgical access is difficult, poses a high risk of permanent neurological damage, resulting in incomplete resection and the tumors are Grades II or III.

Stereotactic radiosurgery (SRS) and fractionated Stereotactic radiotherapy (SRT) have been used in the treatment of symptomatic CSMs for more than 15 years. However, there are very few publications about the long-term disease-free survival rates and monitorization of the neurological abnormalities, radiological findings, and toxicity.

The aim of this paper is to present the results of the treatment with SRS or SRT of 89 patients with Grade I symptomatic CSMs. Efficacy, symptomatology, and toxicity were analyzed using follow-up imaging studies and clinical examinations.

## Patients and methods

A retrospective cohort case study was conducted in the Radiotherapy Department of the Sírio-Libanês Hospital and Beneficência Portuguesa Hospital of São Paulo, Brazil.

This study was previously approved by the Committee on Ethics in Research of our institution. From 1994 to 2009, 89 from 132 patients with symptomatic CSMs were treated with SRS or SRT and followed up during, at least, 3 years. The remainder patients were excluded because they were not able to attend the follow up consultations or the follow up lasted less them 3 years. Previous neurosurgical procedures including the biopsy or resection of tumors were performed in 26 out of the 89 patients (18 patients had a previous biopsy only and 8 patients had attempted resection). Histological confirmation was not required when the diagnosis was based on typical imaging findings (well definition of the lesion dural tail, enhancement with contrast). Seventy-three patients were female (82%) with a median age of 56 years. Fifty-seven patients were treated with SRT and 32 were treated with SRS.

All of the patients with WHO stage II/III were excluded from follow-up, as well as patients who were monitored for fewer than 3 years.

### Treatment protocol

Both SRS and SRT were performed with a 6MV linear accelerator (Brain LAB system). All of the patients were immobilized using an individually formed stereotactic frame and adapted mask system. Patients with tumors larger than 3 cm diameter, with volume higher than 14 cc, or very close to the visual pathways were treated with SRT. The median total dose of SRT was 50.4 Gy (range 45 - 54 Gy) fractionated as median single doses of 1.8 Gy (range 1.8- 2 Gy). The median total dose of SRS was 14 Gy (range13-15 Gy). The doses of SRS or SRT covered, at least, 95% of the tumour volume treated at the 80-90% of the dose curve.

### Follow-up protocol

**Figure 1:**
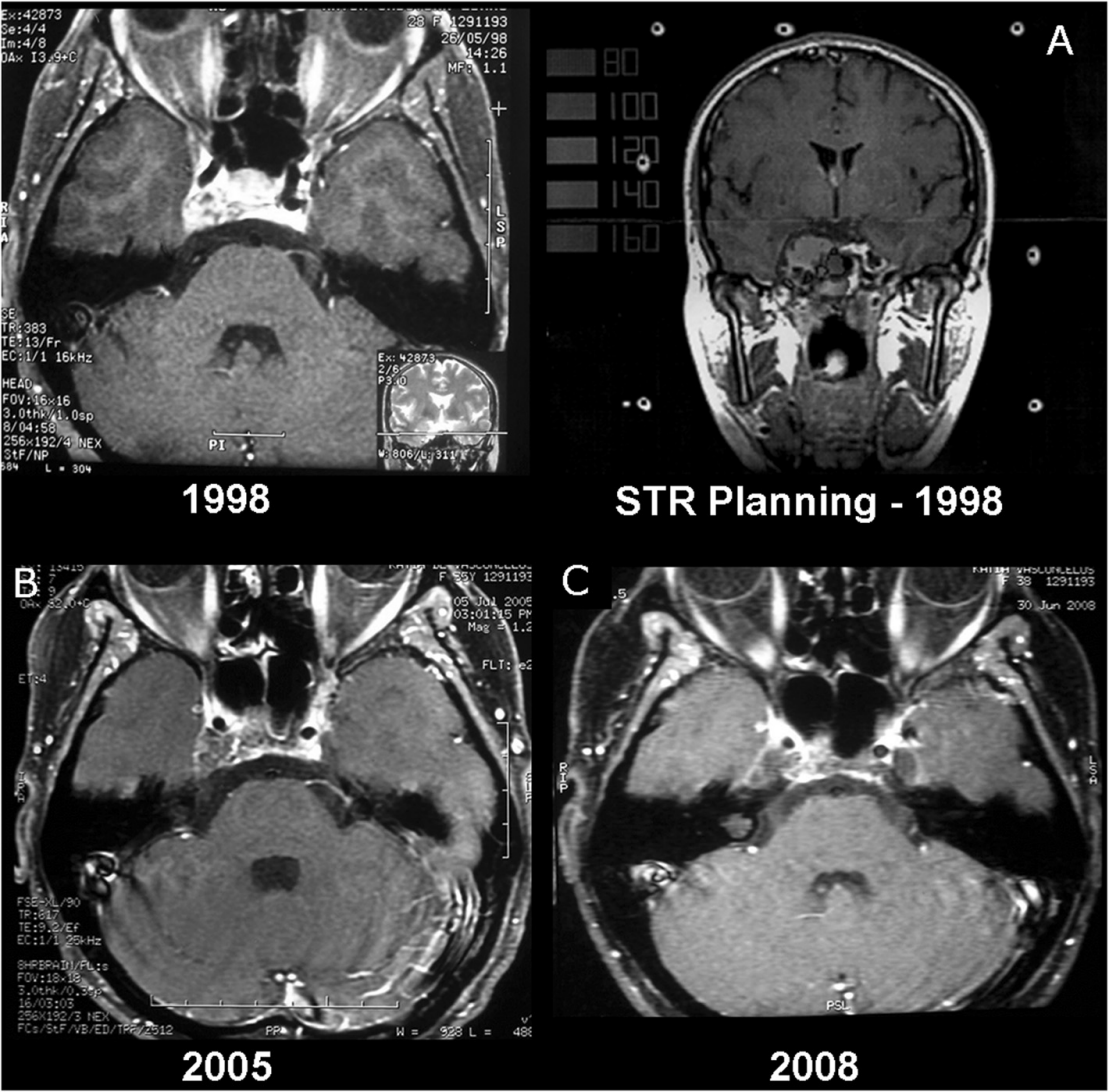
**MR images demonstrating radiological evolution in 10 years by STR treatment of patients with CV meningiomas. A**: MRI for STR planning (1998); **B**: MRI after 5 years of the STR; **C**: MRI after 9 years of the STR.

All of the patients were followed prospectively after the treatment. The protocol included medical evaluations for neurological symptoms and cranial MRI. A first follow-up visit was scheduled 40 days after completion of radiation, and at 3 month intervals thereafter for the first year. From the second year on, follow-up intervals were extended to 6-12 month intervals or as requested clinically. At least 3 years of follow-up was required. Progression free survival was determined based on the RECIST criteria that evaluate two orthogonal diameters of the tumour. Radiological response was defined as the disappearance of the enhancement of the tumour mass or shrinkage of initial volume by at least 20 mm on MRI (Figure 1). Clinical response was defined as regression in signs or symptoms of up to 80% based on pre-treatment levels.

### Statistical analysis

Patient characteristics were described using relative and absolute frequencies. The “performance status” (KPS), gender, age, tumour volume, and duration of symptoms were described by group and compared between treatments using Student’s t-test, with the exception of symptom duration, which was compared using the Mann–Whitney Test.

The presence of each symptom was described using symptom percentages both before and after treatment, compared between treatment time-points, using the McNemar Test.

Improvements in symptoms at the end of treatment were recorded according to the type of treatment, while association of treatment with improvement in individual symptoms was verified using the Fisher’s exact test or the chi-squared test. Kaplan–Meier survival curves were plotted using average times to clinical and radiological improvement by treatment type. Time taken to improvement after each treatment type was compared using the log rank test. A significance level of 5% was adopted for all of the tests.

## Results

### Local tumour control and neurological symptoms

Between 1994 and 2010, 132 patients with symptomatic cavernous sinus meningiomas were treated with SRS and SRT, among whom follow-up was possible in 89 patients. The median follow-up period was 73 months (range: 36-129 months). The patients were predominantly female (82%), and 64% were treated with SRT, whereas 29.2% underwent surgical resection before radiation therapy.

The results summarise the pre-treatment quantitative measures assessed for KPS, duration of symptoms, age and tumour volume. Only tumour volume differed between the two treatment techniques (p < 0.001). The mean duration of symptoms was 18 months (SD = 24 months), the mean KPS was 89.4% (SD = 5.3%) and the mean age of the patients was 58.5 years (SD = 16.1 years) - Table 1.

**Table 1:** Description of quantitative measures assessed by group, and the results of the comparative tests

**Varible**	**Treatment**	**Mean**	**SD**	**Median**	**Minimum**	**Maxumum**	**N**	**p**
KPS (%)	SRS	90.00	5.08	90	80	100	32	0.457
	SRT	89.12	5.44	90	80	100	57	
	Total	89.44	5.30	90	80	100	89	
Duration of symptms (months)	SRS	15.74	23.03	9	3	120	32	0.208*
	SRT	19.04	24.62	12	1	156	57	
	Total	17.86	23.98	11	1	156	89	
Age (year)	SRS	61.03	16.38	55	39	107	32	0.278
	SRT	57.12	15.87	56	7	85	57	
	Total	58.50	16.07	56	7	107	89	
Tumor volume (cc)	SRS	8.25	10.88	6	1.5	58.7	32	<0.001
	SRT	25.39	9.91	23.6	10.9	48	57	
	Total	19.23	13.14	18.2	1.5	58.7	89	

Results based on the Student’s t-Test.

*Results based on the Mann–Whitney Test.

There were confirmation of statistical equality regarding time to clinical and radiological improvement between the treatments (p > 0.05). Isolation characteristics examined in the patients statistically influenced the time to clinical improvement (p > 0.05). Patients older than 60 years exhibited a time to radiological improvement that was statistically lower than for patients who were younger than 60 years (p = 0.002). Mean clinical improvement was approximately 80 months, while the radiological improvement is approximately 70 months. Relapse occurred in only 4 patients, with a mean of 124 months (Table 2).

**Table 2:** Estimates of the median times to clinical and radiological features of interest, as well as the second results of the comparative tests

**Final outcome**	**Variables**	**Estimated average**	**Time standard error**	**CI (95%)**		**N Events**	**N Total**	**%**	**p**
				**Inferior**	**Superior**				
Clinical improvement	**Gender**								0.342
	Female	81.97	5.52	71.15	92.80	29	73	39.7	
	Male	70.62	12.51	46.10	95.13	8	16	50.0	
	**Age (years)**								0.152
	< 60	85.56	6.55	72.72	98.40	18	50	36.0	
	> = 60	71.19	7.97	55.57	86.82	19	38	50.0	
	**Tumor volume (cc)**								0.102
	< 14	72.99	6.78	59.70	86.27	25	51	49.0	
	≥ 14	89.25	7.38	74.79	103.72	12	38	31.6	
	**Treatment**								0.972
	SRS	79.64	8.67	62.65	96.62	13	32	40.6	
	SRT	80.01	6.27	67.72	92.30	24	57	42.1	
	**Total**	**79.95**	**5.08**	**70.00**	**89.90**	**37**	**89**	**41.6**	
Radiological improves	**Gender**								0.639
	Female	68.75	6.12	56.76	80.75	36	73	49.3	
	Male	76.10	12.55	51.50	100.70	7	16	43.8	
	**Age (years)**								**0.002**
	< 60	85.02	6.93	71.45	98.59	17	50	34.0	
	≥ 60	52.54	8.05	36.77	68.31	25	38	65.8	
	**Tumor volume (cc)**								0.990
	< 14	69.93	7.22	55.79	84.08	25	51	49.0	
	≥ 14	69.96	8.57	53.17	86.74	18	38	47.4	
	**Treatment**								0.762
	SRS	68.24	9.26	50.08	86.40	16	32	50.0	
	SRT	71.03	6.87	57.57	84.49	27	57	47.4	
	**Total**	**70.04**	**5.51**	**59.23**	**80.85**	**43**	**89**	**48.3**	
Recurrence		124.49	2.19	120.20	128.79	8	89	8.49	
Log-rank test									

In Table 3, all the symptoms showed a statistically significant improvement after treatment (p < 0.05), with the exception of epilepsy (p = 0.25). However, more than half of the cases of epilepsy disappeared (3 of the 5 cases).

**Table 3:** Description of individual symptoms pre- and post-treatment and results of comparative tests

	**Treatment**				
**Variable**	**Pre**		**Post**		**p**
	**n**	**%**	**n**	**%**	
**Pain: Frontal/Facial/Paresthesia (V PAR-V1/V2)**					**<0.001**
No	32	36.0	86	96.6	
Yes	57	64.0	3	3.4	
**Epilepsy**					0.250
No	84	94.4	87	97.8	
Yes	5	5.6	2	2.2	
**Cognitive/Dysthymic Alteration**					**<0.001**
No	61	68.5	85	95.5	
Yes	28	31.5	4	4.5	
**Decreased visual acuity-AMBLYOPIA**					**<0.001**
No	37	41.6	57	64.0	
Yes	52	58.4	32	36.0	
**Visual fields**					**<0.001**
No	38	42.7	54	60.7	
Yes	51	57.3	35	39.3	
**Proptosis**					**<0.001**
No	64	71.9	88	98.9	
Yes	25	28.1	1	1.1	
**Palpebral Ptosis (III PAR) + Divergent Strabismus)**					**<0.001**
No	46	51.7	75	84.3	
Yes	43	48.3	14	15.7	
**Diplopias**					**<0.001**
No	36	40.4	76	85.4	
Yes	53	59.6	13	14.6	
**Unilateral Mydriasis unreactive to light - Anisocoria**					**<0.001**
No	50	56.2	78	87.6	
Yes	39	43.8	11	12.4	
**Recurrence**					
No			81	91.1	
Yes			8	8.9	
**Total**	**89**	**100**	**89**	**100**	

Results on McNemar test.

The improvement in each symptom was the same regardless of treatment. No statistically significant association was found between treatment type and improvement in individual symptoms (p > 0.05) (Table 4).

**Table 4:** Description of improvement in individual symptoms post- treatment by treatment type and results of association tests

	**Treatment**						
**Variable**	**SRS**		**SRT**		**Total**		**p**
	**n**	**%**	**n**	**%**	**n**	**%**	
**Pain Frontal/Facial/Paresthesia (V PAR-V1/V2)**							0.550
No	32	100.0	54	94.7	86	96.6	
Yes	0	0.0	3	5.3	3	3.4	
**Epilepsy**							0.127
No	30	93.8	57	100.0	87	97.8	
Yes	2	6.3	0	0.0	2	2.2	
**Cognitive/Dysthymic Alteration**							0.131
No	29	90.6	56	98.2	85	95.5	
Yes	3	9.4	1	1.8	4	4.5	
**Decreased Visual Acuity-AMBLYOPIA**							0.820*
No	20	62.5	37	64.9	57	64.0	
Yes	12	37.5	20	35.1	32	36.0	
**Visual Fields**							0.522*
No	18	56.3	36	63.2	54	60.7	
Yes	14	43.8	21	36.8	35	39.3	
**Proptosis**							0.360
No	31	96.9	57	100.0	88	98.9	
Yes	1	3.1	0	0.0	1	1.1	
**Palpebral Ptosis (III PAR) + Divergent Strabismus)**							0.233*
No	25	78.1	50	87.7	75	84.3	
Yes	7	21.9	7	12.3	14	15.7	
**Diplopiaa**							0.363
No	29	90.6	47	82.5	76	85.4	
Yes	3	9.4	10	17.5	13	14.6	
**Unilateral mydriasis unreactive to light- Anisocoria**							>0.999
No	28	87.5	50	87.7	78	87.6	
Yes	4	12.5	7	12.3	11	12.4	
**Total**	**32**	**100**	**57**	**100**	**89**	**100**	

*Result on Fisher’s Exact Test.

*Result on Chi Square Test.

**Figure 2:**
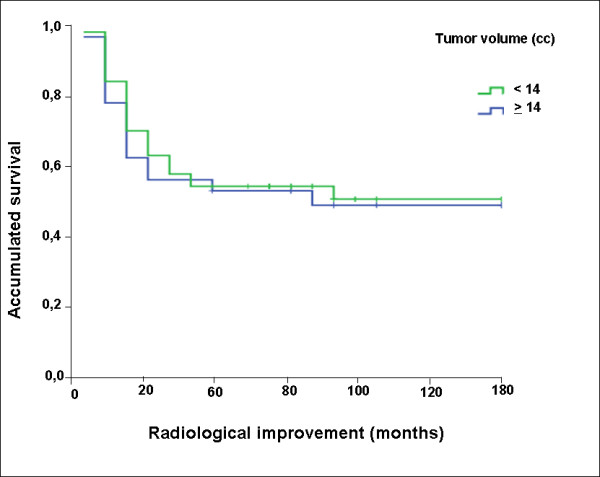
Kaplan-Meier survival curve for the period of time of clinical improvement according to tumor volume of more than 14 cc or less than 14 cc.

No significant difference in time to clinical and radiological improvement was detected between the two types of therapy (p > 0.05) Figure 2. Clinical and radiological improvement was seen in 41.6% and 48.3% of patients, respectively. In the present study, clinical improvement was defined as up to 80% disappearance of the signs and/or symptoms present at pre-treatment. After therapy, approximately 37% of the patients retained at least one neurological complaint that was originally present before radiotherapy.

**Figure 3:**
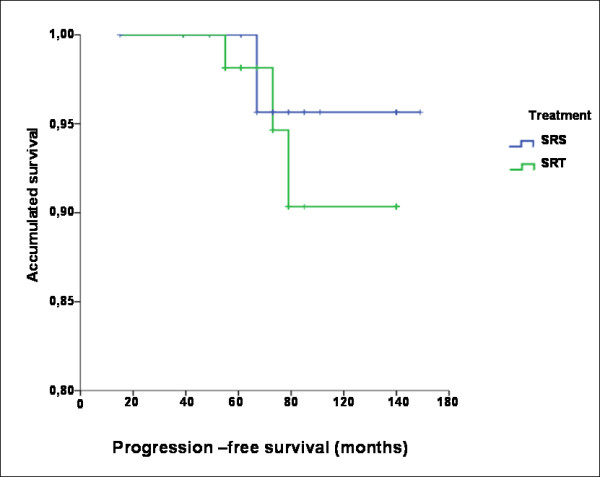
Kaplan-Meier curves for progression-free survival.

For the overall group, progression-free survival after 5, 10, and 15 years was 98.8%, 92.3%, and 92.3%, respectively. For the group treated by SRS, the corresponding disease-free survival rates were 100%, 95.7%, and 90.3%, while for the SRT group, the same rates were 98.1%, 90.3%, and 90.3% (p = 0.567), respectively (Figure 3). No severe complications were seen in the population studied. Disease was recurrent in four patients (4.5%). Among the 89 treated patients, seven experienced temporary morbidity related to SRS and were treated with dexamethasone. Many recovered spontaneously, while two patients had trigeminal neuropathy (CTC grade 2), also regressing rapidly with steroid use. One patient had total occlusion of the internal carotid artery with no neurological repercussions (CTC grade 2).

Lethargy and headache (CTC grade 1) were the most frequently reported immediate symptoms during the treatment.

No fatal treatment complications occurred, although eight patients passed away during the follow-up period. All mortality was due to co-morbidities or older age and not due to tumour recurrence.

Following the SRS/SRT treatments, no radiation-induced malignancies were noted during the 15-year follow-up.

## Discussion

CSMs cause very incapacitating symptoms. The clinical presentations of these tumors are variable and depend on the location and size of the lesion. Fortunately very often they are asymptomatic and slow growing. Headache, diplopia, amblyopia, facial paresthesias, and retroocular pains are also frequent [2]. Unilateral visual deficit, eye proptosis, convulsions, and neurocognitive disturbances (dysthymias) may also occur in our patients [4]. During the last two decades, there was an increasing number of studies evaluating results of primary or adjuvant radiotherapy for treatment of meningiomas, especially when the preservation of the neurological function is critical. Satisfactory disease control and low morbidity rates were observed in many of the series of CSMs patient treated with SRS. However the majority of the papers published about the treatment of CSMs focuses in the context of “lack of progression”. The present study give special attention to the long term clinical and radiological outcomes of symptomatic CSMs patients treated with SRS or SRT.

Nicolato et al. [5] published a retrospective series evaluating 122 benign cavernous sinus meningiomas treated with SRS at a marginal dose of 14.6 Gy. After a median follow-up period of 48.9 months, disease-free progression over five years was 96.5%. Another series published by Lee et al. [6] examined 159 cases of cavernous sinus meningiomas treated with SRS at a marginal dose of 13 Gy; in this series, 49% of cases had undergone previous.

surgical treatment. In those patients with typical meningioma, the control rate was 93.1% over ten years. In the patients who had SRS as the primary treatment, the five-year local control rate was 96.9%. In a similar study, Liscak et al. [7] published a series of 67 cavernous sinus meningioma patients, of whom 64.2% received SRS as the primary treatment. After a median follow-up period of 19 months, no increase in tumour volume was observed in any of the patients. However, these authors reported a temporary morbidity rate of 3.8%.

In tumours greater than three centimetres and near the optic tract, SRT represents a favourable radiotherapy option. Several studies have also shown excellent results in terms of local control (90-100%) with few late complications [8,9].

A study published by Litré et al. [10] examined 100 patients with cavernous sinus meningiomas treated using SRT. Approximately 30% of the patients had undergone partial surgery before this treatment. After a median follow-up period of 33 months, tumour control was 94% in three years. The median dose was 45 Gy, with fractions of 5 Gy given weekly. In terms of symptoms, 81% of the patients with exophthalmia improved, and 52% of diplopic patients noticed improvement, as did 50% of those with cranial nerve V neuropathy. In addition, 67% had improvement in visual acuity. No adverse effects were reported.

Milker-Zabel et al. [11] published a retrospective study in which 57 patients were treated with SRT for meningiomas of the cavernous sinus (29 patients underwent SRT as initial treatment, 10 patients had previous surgery, and 18 patients received SRT after recurrent disease). The median dose was 57.6 Gy, with 1.8 Gy given daily. After a 6.5 year follow up, the tumour control rate was 100%, with overall survival of 95.5% at five and ten years in patients with a WHO Grade I tumour. After a median follow up period of 50 months, Brell et al. [12], noted tumour control of 93% in five years for patients with cavernous sinus meningiomas treated with FSRT. Fifty per cent of these patients exhibited an improvement in neurological symptoms.

These results indicate an improvement in the clinical symptomatology of patients who were treated with radiotherapy (SRT and SRS) ranging from 20% to 69%. This wide variation is most likely related to the heterogeneity of the criteria used in each study, including different definitions for the evaluation of a clinical response. In the present study, a clinical improvement was defined as up to 80% disappearance of the signs/symptoms present at pre- treatment. In all, 41.6% of the evaluated patients responded satisfactorily to treatment, regardless of prior surgical treatment or type of radiotherapy used (SRT or SRS). The radiological response was also very satisfactory, as 92% of the studied patients exhibited attenuation or stabilisation of the disease after treatment. The 15-year progression-free survival was 92.3%, a result that is in agreement with the literature.

With the employment of modern techniques of radiotherapy and its capacity to preserve normal tissue structures, the risk of neurocognitive deficits after treatment is very low. This can be seen clearly in patients who have received adjuvant or rescue radiotherapy after neurosurgery who, nevertheless, do not have a higher rate of cognitive deficits compared to those whose treatment was exclusively surgical [12]. Cognitive deficits are most likely associated with other factors, such as the concomitant use of anti-epilepsy medications and the site of the tumour, and not with the use of radiotherapy [13]. In our series, all cases showed good clinical and cognitive outcomes regardless of previous surgery experience.

## Conclusion

SRT and SRS are safe and reliable techniques for the management of symptomatic CSMs patients. The SRS and SRT allow good local tumor control and improvement of the neurological deficits with reduced complication rate. The 15-year disease-free survival was 92.3% in this group; as such, local control appeared to be excellent at first sight from the perspective of treatment efficacy.

## Competing interests

The authors declare that they have no competing interests.

## Authors’ contributions

We declare that this is an original article and it was never published in another journal. All authors have been involved in analysis, interpretation of data, drafting the manuscript, revising and final approval of the version to be published.
